# Connaissances, attitudes et pratiques des adolescents et des enseignants en matière de contraception: résultats d’une étude qualitative réalisée en République Démocratique du Congo

**DOI:** 10.11604/pamj.2021.38.121.21678

**Published:** 2021-02-03

**Authors:** Dieudonné Mpunga Mukendi, Faustin Chenge Mukalenge, Mapatano Mala Ali, Thérèse Mambu Nyangi Mondo, Gilbert Wembodinga Utshudienyema

**Affiliations:** 1 Ecole de Santé Publique de Kinshasa, Faculté de Médecine, Université de Kinshasa, Kinshasa, République Démocratique du Congo,; 2 Ecole de Santé Publique de Lubumbashi, Faculté de Médecine, Université de Lubumbashi, Lubumbashi, République Démocratique du Congo

**Keywords:** Adolescents, connaissances, attitudes, pratiques, planification familiale, Adolescents, knowledge, attitude, practice, family planning

## Abstract

**Introduction:**

notre objectif était d´analyser les connaissances, attitudes et pratiques des adolescents et des enseignants sur la planification familiale (PF).

**Méthodes:**

en s´appuyant sur la théorie d´action raisonnée publiée en 2011 par Fishbein et Ajzen, une étude qualitative était conduite en 2018 auprès de sept enseignants et 62 adolescents de 15-19 ans. Les données étaient collectées à travers six focus groups (FG) des adolescents et sept interviews semi-structurées des enseignants. Elles étaient analysées à l´aide du logiciel Atlas Ti suivant une approche déductive.

**Résultats:**

l´abstinence périodique, les préservatifs masculins et les pilules étaient les seules méthodes contraceptives citées. Les adolescents et les enseignants avaient de l´appréhension pour utiliser les méthodes contraceptives artificielles en dehors du préservatif masculin utilisé irrégulièrement. Les filles préfèrent les méthodes naturelles, craignant les effets secondaires. La majorité d´adolescents désirent être informés sur la PF à l´école; cependant, ils estiment insuffisant le contenu du cours d´éducation à la vie (EVIE) et fustigent le manque d´ouverture des enseignants. Les pairs, les frères, les sœurs et l´internet étaient les principales sources d´information. Les mères étaient une source importante d´information surtout pour les filles contrairement aux pères en général moins appréciés.

**Conclusion:**

les connaissances sur la PF sont faibles. Les idées fausses sur la contraception entrainent le recours aux pratiques peu efficaces pour prévenir les grossesses non désirées. Pour améliorer ces connaissances, un programme de formation des enseignants devrait être élaboré; le contenu du cours d´EVIE formalisé et réglementé.

## Abstract (en)

**Introduction:**

the purpose of this study was to assess adolescents and teachers´ knowledge, attitude and practices towards family planning (FP).

**Methods:**

in 2018, a qualitative study was conducted among seven teachers and 62 teenagers aged 15-19 years based on the Theory of Reasoned Action published by Fishbein and Ajzen in 2011. Data were collected from six focus group (FG) with adolescents and seven semi-structured interviews of teachers. They were analyzed using Atlas Ti software on the basis of a deductive approach.

**Results:**

periodic abstinence, male condoms and pills were the only contraceptive methods reported. Adolescents and teachers were apprehensive about using artificial contraceptive methods other than irregularly used male condom. Girls prefer natural methods fearing side effects. The majority of adolescents wanted to be informed about FP in school; however, they felt that the content of the Life Education Course (EVIE) was insufficient and that teachers lacked of openness. Peers, brothers, sisters and internet were the main sources of information. Mothers were an important source of information especially for girls, unlike fathers who were generally less appreciated.

**Conclusion:**

knowledge about FP is weak. Misconceptions about contraception lead to the use of ineffective practices to prevent unintended pregnancies. Training programs to improve teachers´ knowledge should be developed and the content of the EVIE course should be formalized and regulated.

## Introduction

L´Afrique subsaharienne porte le plus lourd fardeau des avortements non sécurisés chez les jeunes du monde, un quart survenant entre 15 et 19 ans [1]. Dans les pays aux programmes d´éducation sexuelle et de PF performants, l´incidence de l´avortement est faible, à l´inverse des pays aux lois restrictives, où la culture de prévention et l´accès à la contraception sont moins développés [2]. Une proportion importante d´adolescents devient sexuellement active trop tôt, dans un contexte de faible utilisation de la contraception et d´importants besoins non satisfaits [3]. En Afrique centrale, le manque d´information sur la contraception, le manque d´accès à une source d´approvisionnement et le coût élevé des contraceptifs sont causes des besoins non satisfaits élevés [4]. Les adolescents ont peu conscience des risques sanitaires qu´ils courent en affichant des comportements à risque [5].

L´utilisation des méthodes contraceptives est en augmentation en région africaine [6]. Cependant, les taux d´interruption restent aussi élevés, surtout parmi les adolescentes qui ont en plus un accès limité à ce service [7]. Le faible accès aux services de SSR peut résulter en l´utilisation des méthodes dangereuses et de l´avortement [1]. Le faible niveau d´éducation, la structure familiale perturbée, le faible niveau de revenu [8-10] et les connaissances limitées en matière de santé sexuelle et reproductive (SSR) sont causes de la survenue des grossesses non désirées chez les adolescentes [1], dont l´accès à l´information et aux services de PF sont limités. Les adolescents non scolarisés sont particulièrement vulnérables, faisant des choix moins éclairés [11], aggravés lorsque le niveau d´instruction des mères est faible [12]. Dans certains pays, les programmes d´éducation des adolescents portent surtout les infections sexuellement transmissibles (IST) et le VIH. D´importantes lacunes sont identifiées concernant la prévention de la grossesse, l´utilisation du préservatif, la puberté et la sexualité [13]. Les parents, les agents de santé et les enseignants sont reconnus comme sources fiables d´informations [1]. En pratique, les adolescents s´informent auprès des pairs [1,13] et des membres de famille; les filles se confient à leurs tantes, cousines, camarades et même à la pornographie [1]. Il est démontré que le contrôle parental discriminait davantage le comportement sexuel des adolescents par rapport à la communication sur la sexualité avec les membres de la famille [14]. Les écoles sont une source importante d´information mais sous-utilisée [13]. Il existe une controverse quant à la place qu´occupent les médias dans l´éducation sexuelle des adolescents [13,15]. Le pouvoir de négocier l´utilisation des contraceptifs est limité pour les adolescentes, même celles qui vivent en couple [11]. Les interventions axées sur la participation des parents, des enseignants [1,16] et des enfants, dispensées à l´école au début de l´adolescence peuvent avoir des effets durables sur la réduction des comportements de violence et des relations sexuelles avant l´âge de 18 ans [16]. Les interventions dans le domaine des connaissances peuvent résulter en la création de la demande et l´augmentation de l´utilisation des méthodes contraceptives par les adolescents [3,9], en agissant sur les pratiques ambivalentes et contradictoires qu´ont certains adolescents concernant l´utilisation de contraceptifs [17].

En RDC, un début précoce des activités sexuelles est signalé parmi les adolescents [18, 19], qui tiennent à la confidentialité lors des sessions d´éducation sexuelle [20]. Bien que les adolescentes craignent la grossesse non désirée, elles disposent des connaissances limitées sur la contraception [21]. Les obstacles à l´utilisation des contraceptifs sont la mauvaise communication entre conjoints, les normes socioculturelles défavorables, la peur des effets secondaires et le manque de connaissances sur la PF [22]. La majorité d´adolescents fréquentent l´école. Ceux qui ont entre 15 à 19 ans ayant connu un parcours scolaire normal se retrouvent entre les classes de 3^e^ secondaires et la 2^e^ année des études universitaires. L´éducation scolaire en SSR est assurée à travers le cours d´EVIE dont le contenu n´est pas clairement défini. L´objectif de cette étude était d´analyser les connaissances, attitudes et pratiques des adolescents et des enseignants sur la PF.

## Méthodes

**Figure 1:**
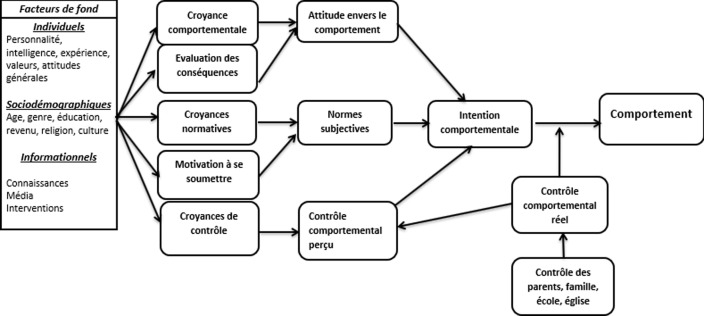
modèle d’action raisonnée de Fishbein et Ajzen, 2011

**Cadre théorique de l´étude:** pour réaliser cette étude, nous avons recouru à la théorie d´action raisonnée, développée par Fishbein et Ajzen en 2011 [23]. Celle-ci contient 4 principales composantes: l´attitude à l´égard du comportement, les normes subjectives, l´intention et le comportement. Cependant, en plus d´agir comme déterminant de l´intention, les contrôles comportemental perçu et réel sont présumés avoir l´influence directe sur le comportement en réduisant le contrôle volontaire sur le comportement (Figure 1). Nous avons supposé que le comportement d´utilisation de la PF pourrait-être planifié par les adolescents dans un contexte régit par des principes contrôlant ces pratiques.

**Type d´étude:** il s´agissait d´une étude qualitative organisée dans la commune de Lemba, à Kinshasa et dans le secteur de Gombe Matadi, province du Kongo Central. Les données étaient collectées à travers six FG des adolescents de 15 à 19 ans et sept interviews semi-structurées des enseignants du cours d´EVIE. Les caractéristiques sociodémographiques de personnes interviewées sont reprises dans les Tableau 1 et Tableau 2. Les adolescents scolarisés étaient identifiés à partir de quatre écoles secondaires sélectionnées par échantillonnage de convenance, à raison de deux écoles à Kinshasa et deux écoles à Gombe Matadi. Nous avons collecté les données auprès des élèves de 3^e^, 4^e^ et 5^e^ secondaire sélectionnés par boule de neige. Les adolescents non scolarisés étaient identifiés par la technique de boule de neige à partir d´un adolescent non scolarisé de chaque sexe, sélectionné à partir de l´église, des associations locales ou d´un informateur.

**Tableau 1:** caractéristiques démographiques des adolescents ayant participé aux groupes de discussion focalisés

	Gombe Matadi		Lemba	
	Garçons	Filles	Garçons	Filles
**Nbre de focus groups**	1	1	2	2
**Nbre des participants**	12	12	24	14
**Groupes d´âge (années)**	15-19	15-17	15-19	15-18
**Statut éducatif**				
3^e^ humanité	3	0	0	0
4^e^ humanité	5	12	11	14
5^e^ humanité	4	0	0	0
Non scolarisés	0	0	13	0
**Religion**				
Eglise de réveil	8	8	9	3
Catholique	0	2	6	4
Protestante	0	0	2	4
Autre	4	2	5	3
Aucune	0	0	2	0
**Tuteur**				
Deux parents	6	7	15	8
Un des parents	2	3	4	2
Autres	4	2	5	4

**Tableau 2:** caractéristiques sociodémographiques des enseignants participants aux interviews semi-structurées

N°	Statut marital	Niveau d´éducation	Ancienneté	Nbre des cours assurés	Nombre d´écoles de prestation
1	Marié	Gradué histoire et sciences sociales	16 ans	2	2
2	Marié	Gradué architecture	1 an	2	1
3	Divorcé	Gradué sciences sociales	9 ans	2	1
4	Célibataire	Etudiant ISP	2 ans	2	1
5	Célibataire	Licencié Economie	5 ans	4	3
6	Marié	Licencié Droit	2 ans	3	1
7	Marié	Gradué biologie-chimie	20 ans	2	1

**Guide d´entretien:** le guide d´entretiens était élaboré en s´appuyant sur le modèle d´actions raisonnées sus-évoqué. Les thématiques abordées ont porté sur les connaissances des méthodes de PF, les sources d´informations en SSR; les pratiques (comportement); les attitudes et préférence (croyances) en matière de contraception; le rôle de l´école et des tuteurs dans l´éducation sexuelle des adolescents.

**Contrôle de qualité et analyse des données:** les données étaient analysées à l´aide du logiciel Atlas Ti en se référant au cadre théorique. A partir des thèmes retenus, nous avons sélectionné des codes ouverts et axiaux. La première partie du processus analytique a consisté à fractionner les données, suivi de l´analyse pour les différences et les similitudes; les concepts similaires étaient étiquetés du même nom. Chaque concept était ensuite défini en termes d´un ensemble de propriétés et de dimensions discrètes pour ajouter de la clarté et de la compréhension. La deuxième partie du processus analytique a traité de la différenciation entre les codes ouverts et axiaux. Les codes ouverts consistaient à mettre en évidence ce qui avait été rapporté plusieurs fois, ainsi que des aspects du comportement pris pour acquis. Avec les codes axiaux, il était possible de créer des nouveaux sous-thèmes en regroupant des phrases et des mots. En utilisant le codage axial, nous avons pu indiquer la manière dont les connexions sont établies entre catégories et sous-catégories ainsi créées. Les thèmes apparaissant dans la section des résultats sont issus de codes ouverts et axiaux. Les résultats ainsi obtenus étaient validés avec les élèves d´une école de la commune de Lemba.

Le protocole de l´étude était approuvé par le comité d´éthique de l´École de Santé Publique de Kinshasa sous le numéro ESP/CE/027/2018. Tous les participants avaient donné un consentement éclairé. L´étude était autorisée par les responsables des écoles.

## Résultats

Les résultats couvrent trois thèmes qui expliquent les obstacles aux initiatives d´amélioration de l´utilisation de la contraception.

### Connaissances et sources d´information sur la contraception

La quasi-totalité d´adolescents connaissent les risques d´une sexualité non protégée. Ils évoquent le risque de grossesse non désirée et des infections sexuellement transmissibles (IST) comme le VIH-Sida. Quant aux moyens de prévention des risques des grossesses non désirées, les adolescents citent l´abstinence périodique, les préservatifs masculins et les pilules contraceptives. Cependant, les adolescents sexuellement inactifs, scolarisés et non scolarisés, avaient une opinion défavorable à l´utilisation des méthodes contraceptives modernes. Un adolescent a déclaré ceci: “*On peut utiliser les préservatifs et d´autres médicaments dont la publicité passe à la télévision pour se protéger contre la survenue des grossesses non désirées mais moi je crois que tout ceci est déconseillé aussi bien pour les garçons que pour les filles, les préservatifs peuvent entrainer la maladie de la prostate, il faut seulement s´abstenir*” **(TM, 16 ans, FG 5)**. Une partie importante d´interviewés pensent que c´est mieux de s´abstenir des relations sexuelles à l´adolescence et d´attendre l´âge adulte ou le mariage pour pratiquer la sexualité. Une adolescente s´est confiée en disant ceci: “*Un mineur est encore irresponsable, il ne sait pas comment se protéger ni comment se prendre en charge en cas de problème; il faut bien se garder et s´abstenir des rapports sexuels*” **(JK, participante 2, FG 2)**.

Les adolescents sexuellement actifs étaient favorables à l´utilisation des méthodes contraceptives naturelles pour prévenir les grossesses non désirées. Une adolescente a déclaré ce qui suit: “*Il faut que la fille sache comment calculer ses périodes d´ovulation, ceci protège mieux que les médicaments et les préservatifs qui peuvent rendre stérile la femme*” **(MN, 18 ans, FG 1)**.

Certains interviewés donnent l´impression que l´abstinence sexuelle était une imposition de l´entourage ou de la société, ce qui se traduit indirectement à travers les raisons superflues, avancées par les adolescents pour justifier l´abstinence. L´âge majeur est considéré par un nombre important d´adolescents comme facteur de protection contre les risques dus aux rapports sexuels non protégés. Les adolescents lient l´âge à la responsabilité des actes posés et leurs conséquences sur le plan sanitaire. Une adolescente scolarisée s´est confiée en ces termes: “*Pour moi, on n´interdit pas aux jeunes d´avoir des rapports sexuels mais on nous demande d´attendre l´âge de 18 ans pour le faire; il y a peu de risque lorsqu´un adolescent pratique la sexualité à partir de 18 ans. Les filles qui ont les rapports sexuels à 15-16 ans ce n´est pas bon, il faut seulement attendre 18 ans car avant cet âge quand la fille va tomber enceinte, le garçon auteur de la grossesse est irresponsable, il va pousser à avorter et cela pose problèmes*” **(NA, 16 ans, FG 3)**. Les méthodes contraceptives, même le plus courantes, sont peu connues de la majorité d´enseignants. La majorité ont cité la méthode d´abstinence périodique et les préservatifs. Les méthodes d´auto-observation, le coït interrompu, le stérilet, les spermicides, les pilules et implants étaient citées par moins d´un interviewé sur trois.

Quant aux sources d´informations en matière de SSR, celles-ci différent selon qu´il s´agit des garçons ou des filles. La plupart des garçons citent l´école, les amis et les médias (internet) comme principales sources d´information en matière de SSR; les filles ajoutent leurs mères, leurs grandes sœurs, leurs amies parmi les sources d´information. La majorité d´adolescents ont déclaré ne pas se confier à leurs pères lorsqu´ils ont besoins d´informations en matière de SSR. Certains se sont butés à une attitude négative de la part de ces derniers. Une adolescente de 17 ans a déclaré ceci: “*Une voisine est tombée enceinte; quand j´ai posé la question à notre papa de savoir comment une jeune fille peut-elle se protéger des grossesses non désirées, papa m´a dit: pourquoi tu veux savoir, pour aller pratiquer?*” **(BK, 4^e^ scientifique)**.

Pour les enseignants, les principales sources d´information des adolescents en matière de SSR sont le téléphone, l´internet, l´école, éducation diffuse, les médias et les films. Les enseignants sont conscients que le cours d´EVIE ne rencontre pas certaines préoccupations soulevées en classe par les élèves. Un enseignant a déclaré ceci: “*L´école ne suffit pas aujourd´hui pour informer les adolescents en matière de PF, c´est surtout la rue, l´éducation de la rue qui influe sur le comportement des adolescents*” **(Enseignant 7, 20 ans d´ancienneté)**. Et un autre d´ajouter: “*le cours d´EVIE n´influence pas beaucoup les adolescents aujourd´hui; c´est plutôt l´éducation diffuse qui a plus d´influence sur les adolescents; l´école donne le cours d´EVIE mais les enfants sont plus informés par le medias, les collègues, et les autres personnes surtout en ce qui concerne les problèmes non abordés pendant le cours; à l´école on est limité mais à la cité c´est plus*” **(Enseignant 4, 2 ans d´expérience)**. L´école est la source d´information préférée par les adolescents, suivi des parents; les prestataires de soins sont préférés surtout par les adolescents non scolarisés. Pour les élèves de 4^e^ et 5^e^ des humanités, le cours d´EVIE ne répond pas à certaines préoccupations en matière de SSR; certains enseignants refusent de répondre à leurs questionnements.

### Attitudes et pratiques face à la contraception

Les adolescents et enseignants interviewés ne sont pas favorables à l´utilisation des méthodes contraceptives, en dehors des méthodes naturelles comme l´abstinence périodique. Un enseignant a déclaré ce qui suit: “*Je ne pense pas qu´un adolescent de 15, 16, 17 ans puisse utiliser une méthode de PF, ils utilisent parfois les condoms pour éviter de mettre au monde*” **(Enseignant N°5, Kinshasa)**.

Ils n´ont pas l´intention d´utiliser les méthodes contraceptives modernes dans le futur ni de les conseiller aux membres de leur famille. Un participant a déclaré: “*Moi personnellement j´utilise la méthode naturelle en calculant la date de menstruation de mon épouse, je n´utilise pas d´autres méthodes comme les préservatifs; à la maison, j´insiste sur l´abstinence, je refuse que les enfants utilisent les préservatifs, sinon ils vont avoir le gout de faire la sexualité; moi leur papa si je vis jusqu´à cet âge c´est parce que je me suis bien conduit dans ma vie, je n´ai jamais eu à faire les rapports sexuels n´importe comment, depuis que j´ai étudié à l´université j´avais la décision d´avoir une seule femme dans ma vie*” **(Enseignant N°7, Kinshasa)**. L´utilisation des méthodes contraceptives est liée aux connaissances et attitudes vis-à-vis de ces méthodes. Un enseignant a déclaré ce qui suit à la question de savoir quelles sont les méthodes contraceptives qu´il connaissait: “*Je connais l´abstinence; je n´ai pas d´idée sur les restes des méthodes; je suis célibataire et je m´abstiens des rapports sexuels; moi personnellement je n´ai jamais utilisé les méthodes contraceptives; ces méthodes c´est pour les mariés, ceux qui vivent en couple*” **(Enseignant N°5)**.

### Contenu du cours d´éducation à la vie (EVIE)

Le ministère d´éducation nationale a la charge d´élaborer le programme du cours d´EVIE. Dans la totalité d´écoles visitées, le programme officiel de ce cours n´a pas été trouvé. Un programme local de formation est élaboré par chaque enseignant en tenant compte de ses connaissances. Un enseignant du Kongo central a déclaré: “*Je n´ai pas de programme officiel du cours, ce que je donne, j´ai fait des recherches; au niveau de l´école on n´a pas ce programme. Dans d´autres écoles, je crois qu´il y a un programme officiel de formation, car cela dépend d´une école à une autre; normalement il y a un programme dans tous les cours. Un cours ne peut pas être donné sans avoir un programme, c´est sûr qu´il doit y avoir un programme. Le ministère doit avoir un programme, pour que toutes les écoles puisent les matières et chaque école peut ajouter quelque chose et non demander à chaque école de former un programme*” **(Enseignant N°4)**. Au cours des échanges avec les enseignants et les élèves, des programmes variés du cours d´EVIE étaient trouvés en fonction des écoles et du niveau. Ce cours est donné de la 1^e^ à la 6^e^ année dans la majorité d´écoles; dans d´autres écoles, il se donne à partir de la 3^e^ année (Tableau 3).

**Tableau 3:** contenu du cours d´éducation à la vie tel qu´élaboré par les enseignants d´une école privée de Kinshasa

Classes de 1^e^ et 2^e^	Classes de 3^e^ et 4^e^	Classes de 5^e^ et 6^e^
On parle beaucoup sur l'introduction générale; on parle de comment aborder une relation amicale pour que cette relation amicale ne puisse pas se transformer en relation amoureuse; on leur donne les conséquences des relations amoureuses (grossesse); comment on peut nettoyer le corps	On montre tout ce qui se passe dans la vie de l´enfant, il doit connaitre le sexe, quelles sont les conséquences, à quel âge on peut tomber enceinte, comment éviter les infections et comment on peut se conduire; comment se préserver surtout pour les filles pour ne pas être beaucoup plus attirante parce qu´elles cherchent leur visibilité surtout à travers leur aspect morphologique	On commence par l´introduction générale, la virginité, la chasteté, le mariage, le divorce, l´importance du cours éducation à la vie; la responsabilité d´un élève face à sa survie
Durée du cours: 45 minutes par semaine et 30 heures pour l´année	Durée du cours: 45 minutes par semaine et 30 heures pour l´année	Durée du cours: une heure par semaine et 35 heures par an

Le contenu du cours d´EVIE selon les adolescents comprend les avortements, la virginité, la chasteté, le VIH Sida, le risque d´accouchement précoce, la sexualité responsable, le mariage. Il s´agit des notions superficielles sur la prévention des grossesses non désirées. La contraception n´est pas expressément abordée en dehors du chapitre sur la chasteté. Certains enseignants trouvent dangereux d´enseigner les notions de PF aux élèves selon leurs déclarations: “*Enseigner la PF est un problème sérieux, il y a des élèves qui connaissent déjà les garçons ou les filles; d´autres ne sont pas encore arrivés à ce stade-là, il faut les apprendre à éviter le sexe, l´abstinence physique, parce que même si on utilise les préservatifs ça peut se rompre et la grossesse peut intervenir. Même l´utilisation des médicaments, moi je ne suis pas d´accord avec ça; moi quand je suis entré à l´université pour étudier, on me disait que toutes les filles sont malades, même si on utilise le préservatif ça peut se rompre et vous attraperez la maladie*” **(Enseignant 6, 2 ans d´expérience)**. Le cours d´EVIE ne bénéficie ni des visites d´inspection du ministère de l´éducation national ni de celles des conseillers pédagogiques des écoles. Un enseignant a déclaré: “*Jusque-là pour le cours d´EVIE, je n´ai pas encore reçu la visite des inspecteurs scolaires depuis que je suis là il y a plus de 20 ans, mais pour les autres cours comme la biologie, la microbiologie, les sciences oui les inspecteurs viennent, ils ont l´horaire*” **(Enseignant N°7, Kinshasa)**. Un autre enseignant a déclaré: “ *Je n´ai pas encore reçu la visite des conseillers scolaires, peut être chez les autres professeurs mais moi, pas encore dans le cadre de ce cours mais pour les autres cours ils entrent; dans le cadre de cours d´EVIE parfois ils regardent dehors en jetant un coup d´œil mais entrer dans la salle pour assister comme visite officielle, non*” **(Enseignant N°6)**.

## Discussion

Les adolescents et les enseignants disposent des faibles connaissances et des idées fausses sur la contraception. Ces résultats indiquent la probable faible vulgarisation de la PF parmi les adolescents et les enseignants. Les idées fausses sur la contraception ne sont pas le seul apanage des adolescents; Lara *et al*. [24] dans une étude réalisée à l´Est de la RDC, ont souligné les idées fausses sur certaines méthodes modernes de contraception de la part des prestataires, des utilisatrices et des membres de la communauté. Yoost *et al*. [25] ont rapporté que même dans les pays développés, les idées fausses persistent concernant l´utilisation du stérilet, particulièrement parmi les jeunes et les femmes nullipares. En dehors de ces observations, l´attitude critique des prestataires de soins et le manque de confidentialité peuvent dissuader l´utilisation de la contraception par les adolescents [26]. Un des obstacles à la prise des contraceptifs par les adolescents est le faible niveau éducation [27] et des connaissances limitées en SSR qui parfois entrainent le recours aux méthodes peu efficaces et même dangereuses de PF [1]. Ces observations sont conformes à celles que nous avons trouvées; les adolescents scolarisés ne reçoivent pas les informations susceptibles de moduler leur attitude en vers la PF. La plupart d´adolescents interviewés ont conscience de l´exposition au risque de grossesse non désirée en cas de sexualité peu contrôlée. Cependant, ce fait contraste avec leur intention négative vis-à-vis des contraceptifs. C´est probablement pour cette raison que, dans un contexte de faible niveau des connaissances sur la contraception, ils préfèrent s´abstenir de tout rapport sexuel ou d´utiliser les préservatifs. Cette prise de conscience est moindre comparativement aux résultats de Krugu *et al*. pour qui les filles qui parlaient de la sexualité avec leur mère et recevaient l´éducation sur l´utilisation de préservatifs à l´école avaient une attitude favorable face aux préservatifs [28]. Cependant, comme cela est aussi indiqué dans notre étude, la plupart d´adolescents ont une attitude négative en vers les autres méthodes de PF [28, 29] et utilisent de manière incohérente ou inconstante les préservatifs [30]. Des déterminants sociaux multi-niveaux tels que les influences interpersonnelles (pairs, partenaires et parents), communautaires (normes sociales) et macro-sociales (la religion, les enseignements sur les rapports sexuels avant le mariage et l´accès limité aux soins de qualité) conditionnent l´utilisation de la PF par les adolescents [29]. La majorité d´adolescents que nous avons interviewé étaient des religions chrétiennes, se confiant à l´entourage (mères, pairs) pour les questions de sexualité. Les adolescents ont la préférence pour leurs parents et l´école pour tout besoin des conseils sur la SSR. L´attitude négative des pères est un obstacle à l´accès à la bonne information. Une communication parentale ouverte sur les questions de sexualité à la maison, une éducation sexuelle complète à l´école et la bonne perception du risque encouru en cas de non utilisation de la contraception constituent des facteurs de protection dans les efforts de prévention des grossesses chez les adolescentes [28]. En cas d´insuffisance d´informations sur la SSR en famille, l´école peut combler ce gap si les enseignements sont coordonnés et suivis par les responsables de l´éducation nationale. Malheureusement, l´école ne fournit pas assez d´informations sur la PF; les connaissances des enseignants sont faibles, le programme officiel du cours d´EVIE n´est pas disponible dans les écoles et les inspecteurs n´en suivent pas le déroulement.

Ces observations poussent à croire que peu d´importance est accordée à l´éducation sexuelle et reproductive des adolescents, pourtant, les conséquences d´une sexualité précoce et non protégée, comme les IST, les grossesses non désirées et les avortements comptent parmi les causes d´absentéisme voire d´arrêt d´études à cet âge [31-33]. La Conférence internationale sur la population et le développement (CIPD) de 1994 avait recommandé de fournir une éducation sexuelle et de soutenir un enseignement et des services de SSR adaptés aux jeunes pour améliorer leurs connaissances en matière de SSR [34]. Cette recommandation n´est pas suivie en RDC. Il est démontré que des liens étroits entre les adolescents et les parents, entre les adolescents et la famille et les liens perçus avec l´école protégeaient les adolescents contre les comportements à risque pour la santé. Une observation indique que l´attente de la réussite scolaire par les parents était associée à des comportements à risque moins élevés pour la santé; de même, la désapprobation parentale pour les débuts sexuels précoces était associée à un âge tardif de début des rapports sexuels [35]. Les adolescentes et les enseignants ont exprimé leur intention d´utiliser les méthodes contraceptives naturelles, connues pour les taux d´échecs élevés en cas de mauvaise utilisation. Selon l´Organisation Mondiale de la Santé (OMS), l´efficacité de la plupart des méthodes naturelles de PF reste assez élevée, variant de 90 à 98% si la méthode est maitrisée et bien appliquée par le couple, la méthode du coït interrompu étant la moins efficace, à 73%. Le succès de l´application des méthodes naturelles dépend largement du niveau d´éducation, des connaissances et pratiques des utilisateurs [36]. Le faible niveau d´éducation et le faible niveau des connaissances sur les méthodes de PF que nous avons trouvé ne garantissent pas une utilisation optimale des méthodes naturelles, bien que préférées par les adolescents. La survenue des grossesses à risque, les naissances non désirées ou les avortements provoqués consécutifs signalés actuellement en RDC peuvent s´expliquer à la suite des observations susmentionnées [18]. Les nombreuses lacunes sur les connaissances de la contraception soulignent le besoin de trouver la meilleure façon de concevoir des interventions efficaces pour adolescents et sur la meilleure manière de les mettre en œuvre [34]. Une des pistes de solution est l´amélioration des connaissances des enseignants en PF et la mise à jour des programmes de formation des adolescents.

Cette étude était conduite dans deux provinces, peu représentatives de l´ensemble de la RDC. Une de ses forces est qu´elle s´est déroulée à Kinshasa, capitale de la RDC et siège de l´administration centrale. Les résultats ont été validés avec les interviewés et une des sous-divisions provinciales en charge de l´éducation.

## Conclusion

Cette étude a montré que les connaissances des adolescents et des enseignants sont insuffisantes en matière de PF. Les idées fausses sur la contraception entrainent le recours aux pratiques peu efficaces dans la prévention des grossesses non désirées. En dehors du fait que l´école et les parents sont de loin préférés comme sources d´informations en PF, le contenu du cours d´EVIE n´est pas consistant. En vue d´améliorer les connaissances en PF, un programme de formation des enseignants devrait être élaboré; le contenu du cours d´EVIE formalisé et réglementé par les responsables de l´éducation nationale.

### Etat des connaissances sur le sujet

- Certains adolescents sont victimes des grossesses non désirées et à risque qui entravent leur vie et celle de leur entourage;
- Les adolescents utilisent moins les méthodes contraceptives comparativement aux adultes;
- Le préservatif masculin est la méthode contraceptive moderne la plus utilisée mais de manière irrégulière par les adolescents.

### Contribution de notre étude à la connaissance

- Les connaissances des enseignants du cours d´éducation à la vie sur les méthodes contraceptives sont aussi insuffisantes que celles des adolescents;
- Les enseignants et les adolescents ont des “idées fausses” sur les effets secondaires de la PF; ils ont une préférence pour les méthodes naturelles de PF;
- Le pauvre contenu du cours d´éducation à la vie est une occasion ratée pour améliorer le niveau des connaissances des adolescents sur la santé sexuelle et reproductive et la planification familiale.

## Remerciements

Nous remercions les responsables du projet de Renforcement Institutionnel pour les Politiques de Santé basées sur les Evidences au Congo (RIPSEC) pour avoir accepté de financer la collecte des données et la publication de cette étude.
